# Implementing a Biomedical Data Warehouse From Blueprint to Bedside in a Regional French University Hospital Setting: Unveiling Processes, Overcoming Challenges, and Extracting Clinical Insight

**DOI:** 10.2196/50194

**Published:** 2024-06-24

**Authors:** Matilde Karakachoff, Thomas Goronflot, Sandrine Coudol, Delphine Toublant, Adrien Bazoge, Pacôme Constant Dit Beaufils, Emilie Varey, Christophe Leux, Nicolas Mauduit, Matthieu Wargny, Pierre-Antoine Gourraud

**Affiliations:** 1 Centre d'Investigation Clinique 1413, INSERM, Clinique des données, Pôle Hospitalo-Universitaire 11: Santé Publique, Centre Hospitalier Universitaire Nantes, Nantes Université, Nantes, France; 2 IT Services, Centre Hospitalier Universitaire Nantes, Nantes Université, Nantes, France; 3 Unité Mixte de Recherche 6004, Laboratoire des Sciences du Numérique de Nantes, Centre National de Recherche Scientifique, École Centrale Nantes, Nantes Université, Nantes, France; 4 l’institut du thorax, Service de neuroradiologie diagnostique et interventionnelle, Centre Hospitalier Universitaire Nantes, Nantes Université, Nantes, France; 5 Direction de la Recherche et de l’Innovation, Centre Hospitalier Universitaire Nantes, Nantes Université, Nantes, France; 6 Service d'information médicale, Centre Hospitalier Universitaire Nantes, Nantes Université, Nantes, France; 7 INSERM Center for Research in Transplantation and Translational Immunology, Nantes Université, Nantes, France

**Keywords:** data warehouse, biomedical data warehouse, clinical data repository, electronic health records, data reuse, secondary use, clinical routine data, real-world data, implementation report

## Abstract

**Background:**

Biomedical data warehouses (BDWs) have become an essential tool to facilitate the reuse of health data for both research and decisional applications. Beyond technical issues, the implementation of BDWs requires strong institutional data governance and operational knowledge of the European and national legal framework for the management of research data access and use.

**Objective:**

In this paper, we describe the compound process of implementation and the contents of a regional university hospital BDW.

**Methods:**

We present the actions and challenges regarding organizational changes, technical architecture, and shared governance that took place to develop the Nantes BDW. We describe the process to access clinical contents, give details about patient data protection, and use examples to illustrate merging clinical insights.

**Implementation (Results):**

More than 68 million textual documents and 543 million pieces of coded information concerning approximately 1.5 million patients admitted to CHUN between 2002 and 2022 can be queried and transformed to be made available to investigators. Since its creation in 2018, 269 projects have benefited from the Nantes BDW. Access to data is organized according to data use and regulatory requirements.

**Conclusions:**

Data use is entirely determined by the scientific question posed. It is the vector of legitimacy of data access for secondary use. Enabling access to a BDW is a game changer for research and all operational situations in need of data. Finally, data governance must prevail over technical issues in institution data strategy vis-à-vis care professionals and patients alike.

## Introduction

The increasing use of electronic health records in research settings presents physicians with the systematic yet secondary use of data collected from multiple sources [1-3]. Indeed, hospital information systems (HISs) face a technical challenge to harmonize and integrate application systems and clinical databases that are highly heterogeneous, are based on editor-specific software formats, and use nonstandardized terminologies [3,4].

Moreover, institutions must face the legal and ethical challenge of granting secondary access to data due to national and international laws vis-à-vis patient privacy [5-7]. The reuse of data produced during the care process implies operational knowledge of ethics and legacy concepts that must be solved through well-defined data governance and access policies [7]. Repositories must ensure not only the technical aspects to data access but also the decision criteria granting access [6]. Indeed, institutional data governance is also pivotal when considering legal and ethical principles such as patient informed consent and privacy data protection [2,8,9]. Last but not least, following the line of traditional epidemiology, clinical data reuse requires treatment within a validated and standardized methodological framework to ensure a qualitative result from a scientific and clinical point of view [9].

Despite these difficulties, the rise of biomedical data warehouses (BDWs) is transforming research processes for epidemiology and clinical studies [5,10-12]. Patient data constitute well-defined profiles that can be used to facilitate the enrichment of cohorts [13,14], patient selection and follow-up for clinical trials [15], phenotypic detection, and detailed descriptions of symptoms. BDWs can facilitate the development and performance of personalized and precision medicine, including through the use of big data and artificial intelligence methods.

The implementation of BDWs is conducted at various geographical levels in France. To our knowledge, 24 active hospital BDWs were set up between 2008 and 2023 [16-21]. Regional coordination often occurs within specialized networks of BDWs such as the “Ouest Data Hub,” which is specifically designed for university hospitals in Western France [22]. This can take place in thematic networks and is well advanced in cancer data [23]. National or European Union–wide initiatives also propose a development and coordination framework to deal with the different challenges of implementation. In particular, France initiated in 2019 a national project called “Health Data Hub” [24,25] that promotes centralized coordination and increases the visibility of data sources on a nationwide level.

In this paper we report our 5-year experience regarding organizational changes, technical architecture, and governance, supporting the implementation of the Nantes University Hospital Biomedical Data Warehouse (NBDW). We describe the process to access clinical content. We also give figures concerning sources and contents included in the repository, and provide some insight into the projects to which the NBDW contributed. Finally, we propose three indicators to measure the effectiveness of the setting up operation.

## Methods

### Overview

In 2018, Centre Hospitalo-Universitaire de Nantes (CHUN; University Hospital of Nantes) implemented a BDW to facilitate secondary use of personal health data originally collected in the context of patient care for research and to offer single secure access to up-to-date data from different sources within the CHUN HIS, accommodating a wide range of data types, including demographic and clinical information, consultations, billing codes, diagnoses, laboratory results, medical notes, and drug administration in a unified view.

The focal point of the intervention geared toward implementing the NBDW entailed orchestrating the reorganization across the involved hospital research entities. This restructuring initiative encompassed not only the resolution of technical hurdles but also the delineation of governance structures, regulatory frameworks, and parameters governing data access.

This implementation report adheres to the iCHECK-DH (Guidelines and Checklist for the Reporting on Digital Health Implementations) reporting guidelines [26].

### From Blueprint to the Functional Organization of the NBDW

**Figure 1.**
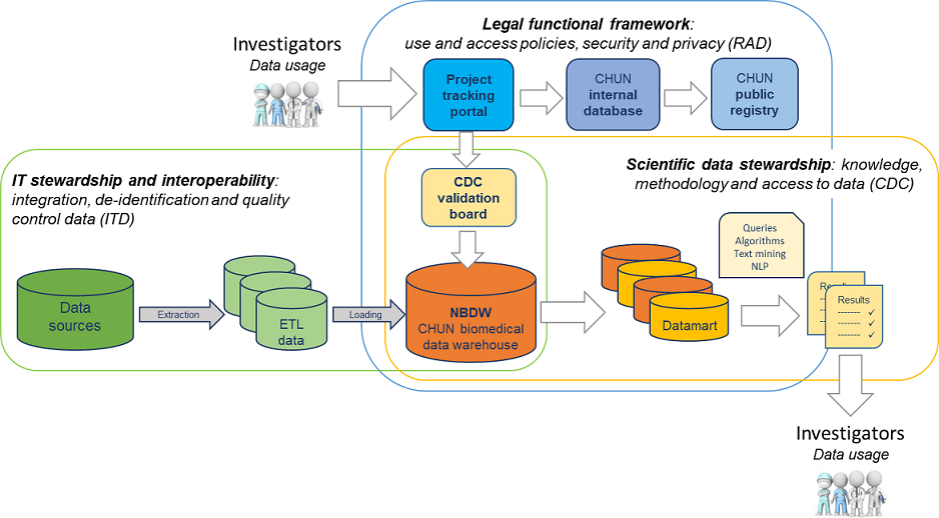
A functional framework dedicated to the CHUN NBDW. A 3-fold governance structure constitutes the functional research framework accompanying the development of the NBDW. The ITD ensures the IT infrastructure’s quality, transparency, and data deidentification. Through an ETL process, data flows are extracted and loaded to the NBDW. The RAD is an administrative department that assumes legal and ethical responsibility, and lays the regulatory framework that will define data use and access policies. The CDC assumes scientific data stewardship, is in charge of scientific responsibility, supports the methodology of the study, and grants data access. Those three structures are organized and structured to ensure research quality, ethics, and technical transparency. White arrows represent the data request and access process undertaken by investigators. CDC: clinical data center; CHUN: Centre Hospitalo-Universitaire de Nantes; ETL: extract, transform, and load; ITD: Information Technology Department; NBDW: Nantes Biomedical Data Warehouse; NLP: natural language processing; RAD: Research Administration Department.

Implementation of the NBDW required the de novo organization of a functional research network within a 3-fold structure (Figure 1). First, enforcement of the governance policy and NBDW legal framework was achieved by the Research Administration Department (RAD), which assumed legal and ethical responsibility. Second, the creation of a clinical data center (CDC) took charge of the scientific question and the potential need for NBDW data to coassume scientific responsibility with project investigators. The last layer of technical responsibility was established by the hospital’s Information Technology Department (ITD). IT priorities are defined by legal and governance constraints, and address the data needs of each project. The new organization allows the distribution of tasks and responsibilities in the implementation and management of the NBDW.

### Legal Functional Framework

On behalf of the hospital, the RAD defines the framework of data use and access policies, security, and patient consent and privacy, and is in charge of the construction, organization, and enforcement of the NBDW regulatory framework. It works in conjunction with the hospital data protection officer, represented by an officer specifically dedicated to research data. Furthermore, the RAD structure requires documented monitoring to ensure a constant adaptation of the NBDW regulatory framework to answer to changes in legal standards. All projects are publicly described on the web [27] for all potential patients contributing data.

### IT Stewardship and Interoperability

The ITD is in charge of the collection, storage aggregation, quality, and integrity of clinical data. This structure carries out a continuous process to conform to technical standards. Setting up the addition of new hospital data sources is an ongoing process that started in July 2018 and is still relevant.

The NBDW is set up on virtual machines. An Oracle database enables massive data storage, integrating multiple hospital data sources into a unique set of tables containing data on patients, consultations, diagnoses, laboratory results, medical notes, and inpatient drug administration. The NBDW uses eHOP [22,28] software to organize and query the database. eHOP is a platform developed by Rennes University using a public-private partnership on the enterprise application integration Enovacom Suite Version 2 (ESV2) of Enovacom (Orange Business Service-Santé). eHOP carries out the acquisition, transformation, and integration of the data coming from various HISs with different formats and standards (Health Level 7 [HL7]; Harmoniser et promouvoir l'informatique médicale [HPRIM]; PN13; Logical Observation Identifiers Names and Codes [LOINC]; and Word documents, PDF, CSV, and text). ESV2 provides an automatic, scheduled (daily, weekly, or monthly), and monitored data supply from the NBDW.

### Scientific Data Stewardship and Mediated Access to BDW

Beginning in 2018, the CDC has had a specific team dedicated to the reuse of health data for research, relying on the expertise of public health doctors, data and computer scientists, epidemiologists, biostatisticians, and project managers promoting epidemiology analyses and providing support to clinical investigators and researchers in data access. In conjunction with the ITD, the CDC defines the standards necessary for data integration and data use practices, and ensures data control and the scientific quality of the analyses.

#### Target and Data Access

The end users of the NBDW are investigators (internal or external to CHUN) aiming to advance the institution’s research. A per-project access is created for CHUN investigators through a standardized approval process consisting of 4 main steps (Figure 1). First, investigators register their request and submit a research protocol through a portal hosted by the CHUN intranet. Second, the CDC validation board, which meets once a week, analyzes three dimensions of the submitted research projects: compliance with ethical principles, scientific relevance, and feasibility. On completion of the approval process, the project is registered on an internal database for the completion of legal requirements. Third, the CDC processes all the necessary queries on the NBDW to select a group of patients relevant to the scientific question. During this step, ongoing collaboration with the investigator is necessary, in particular, to precisely define the scientific purpose, eligible population, and data of interest. Fourth, data are made available internally through a data mart, hosted by the CHUN intranet. The data mart system provides regulated, parsimonious (only the required data regarding the targeted population), and time-limited access to investigators. Data are completely deidentified. Moreover, investigators can make simple queries and seek data on patients contained in the data marts through eHOP, which is enriched with a set of tools for simple textual and structured data queries.

Projects requiring data management and extraction to integrate a research database are declared to the public registry of CHUN projects as a guarantee of transparency and to allow patient opposition. At this step, more complex methods for the extraction of information through natural language processing (NLP) [29], regular expression tools, or other structured data [30] may be applied. Finally, data extraction is constrained to strictly necessary data, following the parsimony principle, and only if access to data can be done in a secure environment.

In the case of a project supported by an external project leader from CHUN (academic or private partner), the same process as described above takes place with the exception of the following differences: the project might be supported by a clinical team that submits the research protocol through the portal; a partnership agreement must be signed between the hospital and the partner, and the data mart is only available through a specific virtual working space (data are still internally hosted).

#### Data Protection and Patient Consent

To comply with national and international privacy regulations, data integration is subjected to a deidentification algorithm. Data are stored in two independent and separate Oracle schemas to separate pseudonymized data from nominative or other directly reidentifying information to which access is strongly limited. Data separation is supplemented by access management and traceability of the actions carried out (ie, AuditLog). Most notably, the platform includes a functionality for collecting and applying patient consent to the use of personal data, ensuring compliance with French law and European General Data Protection Regulation requirements [31].

#### Regulatory Approval

In alignment with the French Data Protection Act (Loi Informatique et Libertés, 1978), the use of personal data for health research and evaluation requires compliance with a reference methodology, representing good practices. Without such compliance, personal data use must be authorized by the Commission Nationale de l’Informatique et des Libertés (CNIL; French National Commission for Information Technology and Civil Liberties). At the launch of the NBDW, no research methodology existed for data warehouses in the field. Therefore, approval from the CNIL was mandatory to initiate implementation. Submission to the CNIL covered legal responsibilities, data processing details, access, governance, and more. Comprehensive data access details were provided, extending to researchers whether affiliated with CHUN or not. Private entities are permitted to engage in research projects based on the NBDW, ensuring adherence to this resolution and French regulations. The Data Protection Impact Assessment for NBDW was an integral part of the submission to the CNIL, serving as a mandatory document. The authorization to set up and use the NBDW was granted on July 19, 2018, by the CNIL (resolution 2018‐295).

#### Budget Planning and Sustainability

Estimating the costs associated with implementing and maintaining a data warehouse is challenging owing to several factors. First, the NBDW is part of an institutional strategy, making it difficult to consider it as a stand-alone entity. Second, implementing it involves the collaboration and coordination of multiple structures and experts, complicating the estimation of resource use. Third, hidden costs are difficult to anticipate and consider, including system failures and delays, unplanned license renewals and upgrades, adjustments to regulatory and legal requirements, unplanned changes in HISs, and infrastructure upgrades. We made an estimation by considering three budget lines—infrastructure, license, and human resources—and two different periods—completion in 5 years (2018‐2022) and maintenance in 2 years (2022 and 2023)—for a total of €2.6 million (US $2.8 million).

In terms of sustainability, an annual operational budget is allocated for maintenance and updates. Specific needs for the integration of new hospital data sources are financed through project-based funding. Moreover, an economic model is currently being defined to incorporate additional charges for infrastructure costs in the case of external research projects.

#### Ethical Considerations

An ethics statement is included in the regulatory approval granted by CNIL with resolution number 2018‐295 [32].

## Implementation (Results)

### Description of the Sources, Concepts, and Contents

The NBDW integrates multiple hospital data sources into a unique and structured set of tables containing data on patient demographic and administrative records, inpatient drug administration, inpatient constants and anthropometric scores and metrics, anatomic pathology notes, inpatient and outpatient medical laboratory results, narrative medical notes (including admission/discharge summaries, inpatient anesthesia notes, outpatient consultation notes, nurse notes), *International Classification of Diseases, 10th Revision* (*ICD-10*) and French Classification Commune des Actes Médicaux (CCAM; Common Classification of Medical Procedures) codes for inpatient diagnoses and procedures, and medical imaging reports. Table 1 shows principal concepts and contents according to different HIS data sources or software integrated up to now. Some data sources contain only narrative notes, and some sources contain both unstructured and structured data.

**Table 1.** Nantes University Hospital Biomedical Data Warehouse principal sources and concepts. Data extracted May 10, 2023.

Concepts	Software	Period	Patients, n	Documents, n	Documents in 2022, n	Structured data, n
Inpatient drug prescriptions	MILLENNIUM	2015-today	318,456	39,681,513	5,673,000	248,289,146
Cardiology narrative notes	CARDIOREPORT	2015-today	9278	38,041	6644	—^a^
Consultation clinical narrative notes	GAM-CLINICOM	2002-today	1,053,386	6,507,860	22,982	—
Constants and anthropometric data	MILLENNIUM	2015-today	440,250	3,306,589	638,969	61,142,757
Anatomic pathology notes	DIAMIC	2015-today	131,812	229,081	27,244	1246
Biology laboratory results	DXLAB	2012-today	701,804	10,752,384	1,672,218	155,825,060
Clinical narrative notes	MILLENNIUM	2015-today	569,114	3,967,073	907,573	5,395,233
*ICD-10*^b^ and clinical procedure codes	CLINICOM	2006-today	725,802	5,318,712	401,607	105,246,620
Radiology reports	QDOC	2015-today	284,937	904,625	122,900	—
Nurse transmissions	TRANSMISSIONS	2017-today	131,508	1,546,114	320,293	1,546,114

a Not applicable.

b *ICD-10*: *International Classification of Diseases, 10th Revision.*

### NBDW Figures and Populations

CHUN, a tertiary care hospital ranked seventh in France in terms of activity [33], provides care over a population catchment area of 1.4 million inhabitants. It provides follow-up and long-term health care for both in- and outpatients. It has 2993 hospital beds, delivers 4380 babies, and conducts more than 1 million consultations and external medical procedures per year [34]. It also carries out practical teaching for 1200 medical students, 800 medical residents, and over 2000 non–medical students.

The NDBW includes information on approximately 1.5 million patients admitted between 2003 and 2022 (Table 2). More than 1.2 million hospitalizations are associated with approximately 12.3 million *ICD-10*–coded diagnoses and 7.3 million clinical procedure codes. Together with more than 6.3 million external consultations, the NBDW contains more than 11 million textual documents. These narrative notes integrated as free-text documents can be interrogated and turned into structured data for research.

The yearly number of patients, hospitalizations, consultations, and narrative notes has increased over time (Multimedia Appendix 1) with growth rates between 2003 and 2019 ranging from 109% (hospitalizations) to 430% (outpatient consultations).

**Table 2.** Nantes University Hospital Biomedical Data Warehouse figures and contents, 2003‐2022.

Contents	Since 2003, n	2022 only, n
Patients^a^	1,597,498	300,804
Hospitalizations^b^	2,635,809	183,361
Outpatient consultations	6,358,271	524,948
Clinical narrative notes^c^	11,634,761	826,670
Diagnoses^d^	12,251,148	956,688
Clinical procedures^e^	7,272,346	504,571

a Patients with ≥1 clinical narrative note or a structured document, including inpatient and patients admitted for outward consultations.

b Inpatient hospitalizations in medical, surgical, and obstetric services, including complete hospitalizations, day-hospital admissions, and recurring visits.

c Clinical narrative notes (with the exclusion of vital signs and anthropometric data; *International Classification of Diseases, 10th Revision* [*ICD-10*] and clinical procedure codes; laboratory results; inpatient drug administrations; and nurse transmissions).

d Medical diagnoses following the *ICD-10* for medical, surgical, and obstetric hospitalizations.

e Classification Commune des Actes Médicaux for medical, surgical, and obstetric hospitalizations.

### Projects and Effectiveness Outcomes

The availability of NBDW data makes it possible to provide data in response to a wide array of scientific questions and the need for data in the analysis and management of care and organization. Prior to the creation of NBDW data, researchers were limited to the interrogation of medico-administrative–structured information out of the scope of data reuse consent. It only covered structured data such as *ICD-10* diagnoses associated with hospitalizations; medical procedures through CCAM codes; and, to a lesser extent owing to availability issues, laboratory results. Obtaining results from different queries performed independently on different HISs and data manager services was time-consuming if not impossible for both legal and technical reasons.

The NBDW currently facilitates queries of clinical concepts in both structured and unstructured free-text notes in an integrated environment and with respect to data protection policies and laws. BDW data requests may be divided into three different types of research projects according to their purpose: (1) optimize patient screening in both clinical trials and observational studies, (2) enrich case reports or electronic case report forms for disease surveillance, and (3) evaluate and improve clinical practices and resource management. To illustrate different types of studies, three concrete examples of data use are described in Multimedia Appendix 2 [35-37].

The first outcome to measure the effectiveness of the NBDW was defined as the number of studies supported through the project tracking portal (Figure 1). Since 2018, 577 requests have been made and treated by the CDC. Among them, 269 projects involved patients included thanks to NBDW queries and research tools (second outcome), and for 115 of them, data marts were created to give investigators access to data (third outcome).

## Discussion

### Lessons Learned

The development of clinical data warehouses has provided unprecedented access to a large amount of diverse data from clinical care. However, it requires a dedicated effort in terms of governance, data access rules with respect to patient consent and data protection, and technical challenges. The reorganization of structures within a functional research framework is the first factor in the success of the NBDW. Collaboration between departments has not only facilitated seamless communication but also engendered the innovation necessary to deal with the complexities of health care data management. It was an opportunity to test new IT technologies such as distributed infrastructure and anonymization techniques such as deidentification. The interaction between structures has also been a fundamental element in the process of obtaining BDW authorization from the CNIL. Indeed, the obtention of regulatory approval is the result of a long negotiation that required expertise in addressing legal, technical, and scientific research requirements (see Regulatory Approval section for more details).

The creation of the CDC, the result of multidisciplinary teamwork composed of computer scientists, NLP engineers, statisticians, physicians, epidemiologists, and project managers, has probably been the second factor of success. The weekly CDC validation boards, supported by the RAD and ITD structures, verify project compliance vis-à-vis three aspects: ethics, scientific relevance, and practical feasibility, giving support and access to the NBDW in a secure context. However, it is also a leading driver in the shift toward data-driven governance in hospitals.

Implementation of the NBDW has required a continuous and still relevant process to conform to new regulatory and technical standards, and to add new hospital sources and ongoing improvements. An important lesson learned was that each of the 269 NBDW projects in the past 5 years has been an opportunity to revisit the contents of the BDW, further the quality control process, and lead the data transformation process, creating data use value.

Establishing networks and working together is probably the best lesson learned. In France, some experiences have led to changes in health data foresight [28] and promoted the implementation of interregional [22] and national hubs. The NBDW has benefited from and contributed to the “Ouest Data Hub,” a network of Western France university hospital data warehouses. The aim is both to facilitate the reproducibility of data analyses, share resources and best querying practices, and promote adapted and standardized terminologies and nomenclatures between centers. Moreover, BDWs use the same software for both integration and querying, allowing them to be interrogated using consistent queries and rules. This approach ensures a high level of interoperability and accessibility, facilitating seamless interaction and adherence to the Findable, Accessible, Interoperable, Reusable (FAIR) principles.

In hindsight, if this process were to be revisited, there are certain facets that we would address differently. Specifically, in the initial stages of implementing the NBDW, primary emphasis was placed on deploying the software and IT infrastructure recommended by the regional network, ODH, aimed at facilitating the establishment of the repository and its querying system. While acknowledging the benefits inherent in this strategy, a more thorough examination of alternative IT solutions is warranted to mitigate reliance on a singular approach and to streamline potential transitions to alternative and variated options.

### Conclusions

In conclusion, conducting health studies using electronic health records requires careful attention to ensure accurate results owing to a lack of a systematic quality process. The data quality control procedure is a long and necessary process [17,38,39]. The future challenge will be the setting up of standardized and shared quality control pipelines to ensure quality results, not only at the local level but also in a regional and national context of future data sharing. By extending long-term investments in IT and data in care institutions, the development of NLP and text-mining tools will further accelerate the use of BDW, facilitating data-driven decision-making discussions from top management to patients.

Any research project and analysis of care-based research performed in health management institutions could benefit from the deployment of organized data access. The collaborative nature of data production and the information and privacy protection for patients require mediated and expert access to the BDW. It demonstrates that technical solutions are partial answers to better data-driven practices and must lead to a clear governance strategy

## Supplementary material

10.2196/50194 Multimedia Appendix 1 Nantes University Hospital Biomedical Data Warehouse yearly data volume by type of data.

10.2196/50194 Multimedia Appendix 2 Three projects as an example of case experiences based on the Nantes University Hospital Biomedical Data Warehouse.

10.2196/50194 Checklist 1 iCHECK-DH (Guidelines and Checklist for the Reporting on Digital Health Implementations).

## Abbreviations

BDW: biomedical data warehouse
CCAM: Classification Commune des Actes Médicaux (English: Common Classification of Medical Procedures)
CDC: clinical data center
CHUN: Centre Hospitalo-Universitaire de Nantes (English: University Hospital of Nantes)
CNIL: Commission Nationale de l’Informatique et des Libertés (English: French National Commission for Information Technology and Civil Liberties)
ESV2: Enovacom Suite Version 2
FAIR: Findable, Accessible, Interoperable, Reusable
HIS: hospital information system
HL7: Health Level 7
HPRIM: Harmoniser et promouvoir l'informatique médicale
*ICD-10*: *International Classification of Diseases, 10th Revision*
iCHECK-DH: Guidelines and Checklist for the Reporting on Digital Health Implementations
ITD: Information Technology Department
LOINC: Logical Observation Identifiers Names and Codes
NBDW: Nantes University Hospital Biomedical Data Warehouse
NLP: natural language processing
RAD: Research Administration Department
