# Supplementary material for: Implementing a Biomedical Data Warehouse From Blueprint to Bedside in a Regional French University Hospital Setting: Unveiling Processes, Overcoming Challenges, and Extracting Clinical Insight
Source: JMIR Med Inform. 2024 Jun 24;12:e50194. doi: 10.2196/50194 (PMC11217163; doi:10.2196/50194)
Supplement: Multimedia Appendix 1 [file medinform-v12-e50194-s001.docx]

**Sup mat 1: CHUN biomedical data warehouse – yearly data volume by type of data**

**
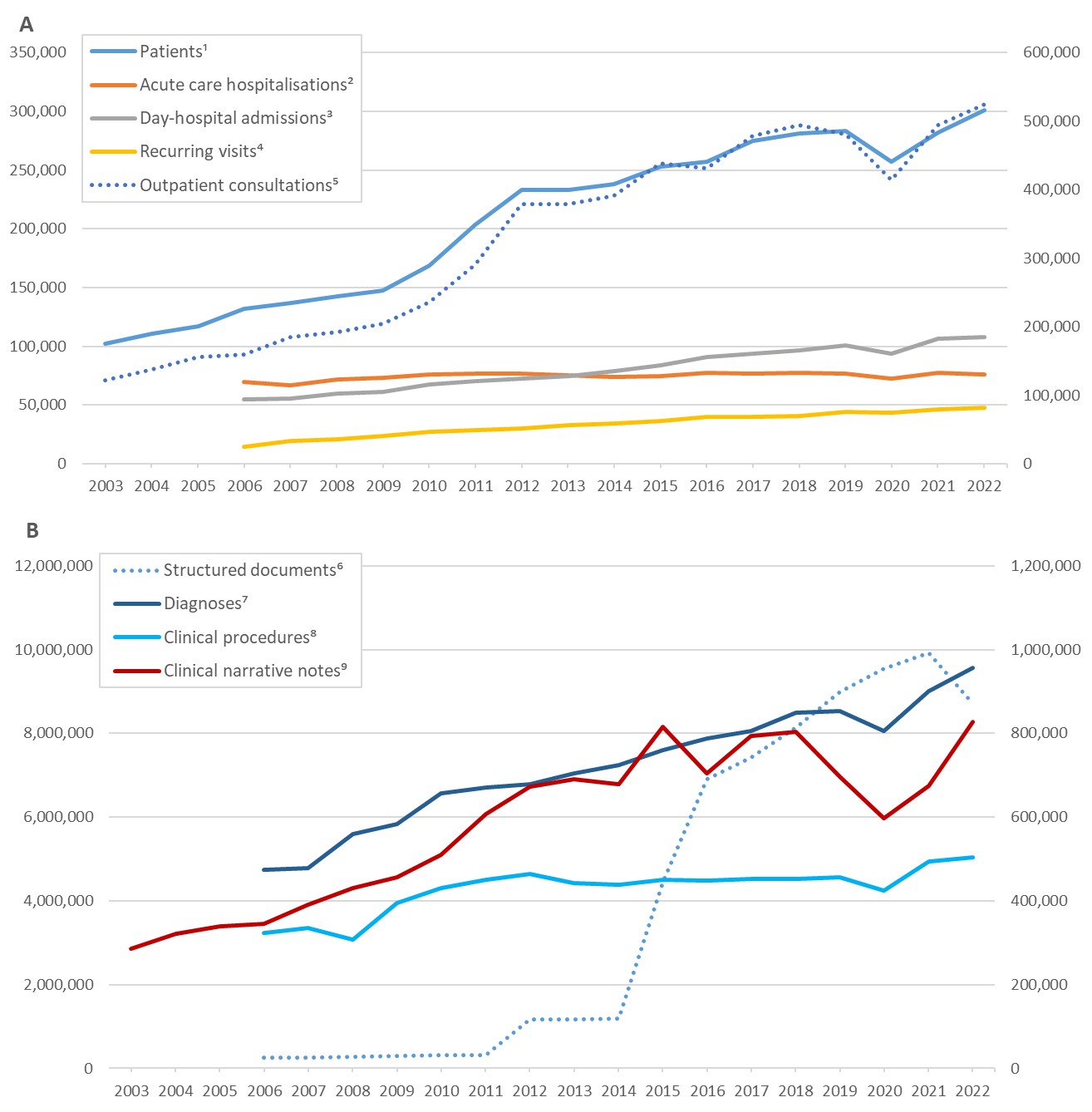
**

Yearly numbers of (A) patients, acute care hospitalizations, day-hospital admissions, recurring visits and outpatient consultations and (B) clinical narrative notes, diagnoses, clinical procedures and structured documents. ^1^Patients with ≥1 clinical narrative notes or structured documents, including inpatient and patients admitted for outward consultations; ^2^Inpatient acute-care complete hospitalizations in medical, surgical and obstetric services; ^3^Inpatient day-hospital hospitalizations in medical, surgical and obstetric services; ^4^Consultations for recurring care: regularly scheduled interventions in medical, surgical and obstetric services; ^5^Outpatient consultations; ^6^Structured documents, including vital signs and anthropometric data, ICD-10 and clinical procedure codes, biology-laboratory results, inpatient drug prescriptions and nurse transmissions; ^7^Medical diagnoses following the ICD-10 associated with hospital stay (both acute care and day-hospital): principal, related and associated diagnoses, of medical, surgical and obstetric hospitalizations; ^8^French Common Classification of Medical Procedures – CCAM codes for medical, surgical and obstetric hospitalizations; ^9^Clinical narrative notes, considered as free-text unstructured documents, out of overall documents included in NBDW. Some data flows (2, 3, 4, 7 and 8) started in year 2006. Starting year 2002 was deemed irrelevant and excluded from the plot. Flows 5, 7, 8 and 9 follow the scale of the secondary (right) axis. CHUN: University Hospital of Nantes; ICD-10: International Classification of Diseases, 10^th^ revision
