# Supplementary material for: Implementing a Biomedical Data Warehouse From Blueprint to Bedside in a Regional French University Hospital Setting: Unveiling Processes, Overcoming Challenges, and Extracting Clinical Insight
Source: JMIR Med Inform. 2024 Jun 24;12:e50194. doi: 10.2196/50194 (PMC11217163; doi:10.2196/50194)
Supplement: Multimedia Appendix 2 [file medinform-v12-e50194-s002.docx]

**Sup mat 2: Three projects as an example of case experiences based on the NBDW**

#### Example 1 - Accelerating screening of control groups, with the identification of patients without intracranial aneurysm

Prediction of intracranial aneurysm (IA) risk is considered as a major issue in public health. Large studies are required in order to encompass power lacking problems as well as to improve performance. ICAN [1] is a French retrospective study, collecting clinical and radiological features on patients harboring IA. In this example, we used the NBDW to select a group of IA-free patients to use as a control group vs. ICAN patients in a case-control study. First, patients who had a brain magnetic resonance imaging (MRI) with time-of-flight angiography on the radiological record were screened, identifying 8,801 individuals. Then, we excluded all patients with a suspected or confirmed IA, through ICD-10 and CCAM IA-related codes (n=4,309 individuals with IA related codes and 1,471 individuals with ICAN exclusion criteria). Individuals with ICD-10 codes containing IA-related diagnoses such as “Elhers-Danlos syndrome”, “mycotic IA”, “fusiform-shaped IA”, “dissecting IA”, “polycystic kidney disease”, “Marfan syndrome”, “fibromuscular dysplasia”, and/or “moya-moya angiopathy” were also excluded (n=278). A group of 5,732 patients without IA was obtained among which an automated procedure to select a random sample matched with ICAN individuals was applied. Thus, NBDW use made it possible to quickly obtain a list of patients meeting general requirements for inclusion in the study. To validate this procedure, a sample of 300 narrative notes was randomly selected and a neurologist was asked to validate the information about the presence or absence of aneurysm through a case-by-case assessment. The algorithm made it possible to correctly classify 36 of 37 patients previously defined as IA patients and 6 of 7 patients without IA, obtaining a TPR at 85.7% (95% confidence interval (CI) [82-90]), a PPV at 75% (95%CI[70-80]), a NPV at 97% (95%CI[95-99]) and a F1 score at 80% (95%CI[75-85]).

#### Example 2 – Data base enrichment, the integration of additional diagnoses and biological information to the French OFSEP Cohort

Multiple sclerosis (MS) is the most prevalent chronic inflammatory and demyelinating disease of the central nervous system, leading to early disability. OFSEP [2] is an in-/outpatient MS French cohort and one of the most complete and broad in the world. In this example, NBDW was used to improve the phenotyping MS OFSEP patients. We found that among the 2,245 patients included in the Nantes cohort, 1,777 had biological data and 1,109 a brain MRI. For those patients, we also found 23,104 CCAM codes, 97,003 ICD-10 diagnoses, 592,210 biological results, 251,531 inpatient drug administrations and 467,462 narrative notes. These data will be structured and reused to improve patient profiling, adding information about areas not previously covered in research. We used the expanded disability status scale (EDSS) as a data quality proxy. In the histogram below, the distribution of the EDSS extracted from the NBDW is set against and compared with the same information from the OFSEP database.

**EDSS as a data quality proxy, comparing distributions in OFSEP and NBDW databases.**


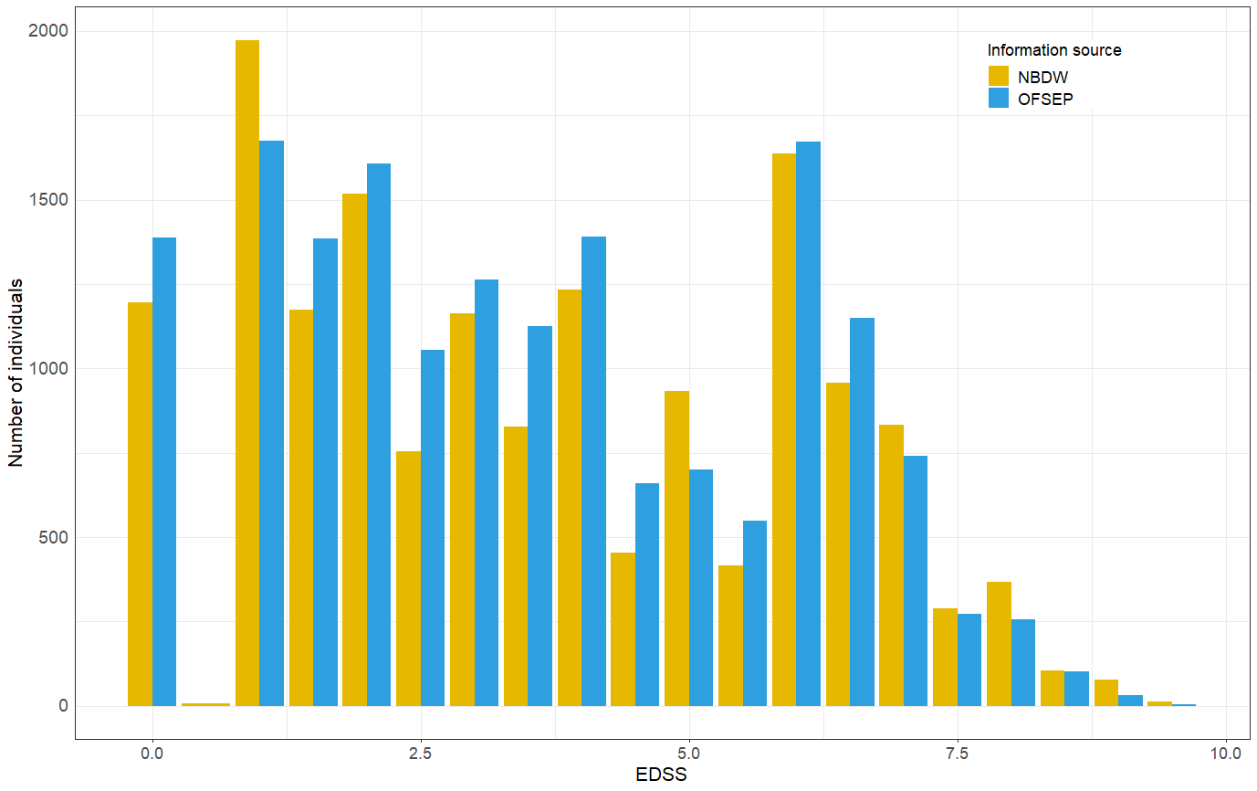


EDSS: Expanded disability Status Scale; OFSEP: French Multiple Sclerosis Observatory (*Observatoire Français de la Sclérose en Plaques*); NBDW: Nantes Biomedical Data Warehouse

#### Example 3 - Application of natural language processing (NLP) tools to monitor a performance indicator.

Standard protocols have been developed by obstetric services to characterize emergency cesarean sections [3]. In this third example, the investigator looked for a global count of unscheduled cesarean sections in 2018, identifiable by color codes in the clinical reports. Specifically, we looked for the report of “red codes”, defined as: emergency procedure label used for maternal shock, collapse encompassing shock, massive antepartum or postpartum hemorrhage, uterine rupture, uterine inversion, sepsis, maternal collapse, obstetric embolism, or eclampsia. The aim of this work was to monitor this indicator annually to see whether the incidence of red codes was maintained below the target set by the team. First, the NBDW was used to identify clinical reports for cesarean section through their title and care unit. We found 934 records that matched the number of unscheduled cesarean sections operated by practitioners. Then, the reports were screened using regular text expressions to extract the color code and compute their incidence. Of all the unscheduled cesarean section clinical reports, 59 red codes (6.3%) were found and verified by surgeons during manual chart review. Thus, the NBDW and NLP tools were useful to automatically generate an indicator of performance of the obstetric unit.

##### Methods

Algorithm performances in example 1 were evaluated using true positive rate (TPR, i.e. recall), positive predictive value (PPV, i.e. precision) and F1-score (computed as the harmonic mean of precision and recall, this score provides a balanced measure of algorithm accuracy), as defined below:

TPR = $\frac{True positive}{True positive+False negative}$

PPV = $\frac{True positive}{True positive+False positive}$

F1-score = $2 \times\frac{TPR * PPV}{TPR +PPV}$

##### References

1. R. Bourcier *et al.*, “Understanding the Pathophysiology of Intracranial Aneurysm: The ICAN Project,” *Neurosurgery*, vol. 80, no. 4, pp. 621–626, Apr. 2017, doi: 10.1093/neuros/nyw135.

2. F. Cotton, S. Kremer, S. Hannoun, S. Vukusic, and V. Dousset, “OFSEP, a nationwide cohort of people with multiple sclerosis: Consensus minimal MRI protocol,” *J. Neuroradiol.*, vol. 42, no. 3, pp. 133–140, Jun. 2015, doi: 10.1016/j.neurad.2014.12.001.

3. D. N. Lucas *et al.*, “Urgency of caesarean section: a new classification.,” *J. R. Soc. Med.*, vol. 93, no. 7, pp. 346–350, Jul. 2000.
